# Leptospirosis incidence and mortality: a spatio-temporal analysis, Ceará, 2007-2023

**DOI:** 10.1590/S2237-96222026v35e20250108.en

**Published:** 2026-05-10

**Authors:** Isabella Martins Camelo, Bruno Victor Barros Cabral, Davi Gomes Sousa, George Jó Bezerra Sousa, Maria Lúcia Duarte Pereira

**Affiliations:** ¹ Universidade Estadual do Ceará, Centro de Ciências da Saúde, Fortaleza, CE, Brazil Universidade Estadual do Ceará Centro de Ciências da Saúde Fortaleza CE Brazil; ² Ministério da Saúde, Brasília, DF, Brazil Ministério da Saúde Brasília DF Brazil

**Keywords:** Spatial Analysis, Weil Disease, Epidemiology, Leptospirosis, Spatio-Temporal Analysis, Análisis Espacial, Enfermedad de Weil, Epidemiología, Leptospirosis, Análisis Espacio-Temporal

## Abstract

**Objective:**

To analyze the spatio-temporal pattern of leptospirosis incidence and mortality in Ceará between 2007 and 2023.

**Methods:**

This was a descriptive observational study with temporal (joinpoint regression), spatial (Bayesian smoothing and Getis-Ord Gi*), and spatio-temporal (scan) analyses to identify risk areas, based on secondary data from the Department of Informatics of the Brazilian National Health System. The study assessed the incidence and mortality rates of leptospirosis in Ceará between 2007 and 2023.

**Results:**

A total of 1,319 leptospirosis cases were recorded, mostly among men (83.9%, n=1,107), young adults aged 15-39 years, and Brown (Brazilian mixed race) people (78.5%, n=1,035). Temporal analysis of incidence revealed three periods of interest: 2007-2009, 2009-2013, and 2013-2023, with respective annual percent changes (APC) and 95% confidence intervals (95%CI): (i) 91.4% (95%CI 24.9; 203.8; p-value 0.002); (ii) -38.4% (95%CI -54.5; -26.8; p-value 0.002); and (iii) 9.4% (95%CI 2.8; 21.1; p-value 0.007). Mortality also showed three distinct periods: a decrease between 2007 and 2014 (APC -13.7%; 95%CI -38.5; -2.8; p-value 0.020), an increase between 2014 and 2019 (APC 27.6%; 95%CI 8.5; 88.8; p-value 0.010), and another decline between 2019 and 2023 (APC -20.6%; 95%CI -58.8; -6.4; p-value 0.010). Hotspots of incidence were identified in Fortaleza, Várzea Alegre, and Pacoti.

**Conclusion:**

Leptospirosis in Ceará is associated with heavy rainfall and precarious socio-environmental conditions. Integrated prevention measures, such as improved sanitation and rodent control, are crucial for reducing the disease burden in the state.

## Resumen

**Objetivo:**

Analizar el patrón espacio-temporal de la incidencia y la mortalidad por leptospirosis en Ceará entre 2007 y 2023.

**Métodos:**

Estudio observacional descriptivo con análisis temporal (regresión de puntos de inflexión), espacial (suavización bayesiana y Getis-Ord Gi*) y espacio-temporal (escaneo) para identificar áreas de riesgo, a partir de datos secundarios del Departamento de Informática del Sistema Único de Salud de Brasil. El estudio evaluó las tasas de incidencia y mortalidad por leptospirosis en Ceará entre 2007 y 2023.

**Resultados:**

Se registraron en total 1.319 casos de leptospirosis, principalmente en hombres (83,9%, n=1.107), adultos jóvenes de 15 a 39 años, y personas clasificadas como pardas (78,5%, n=1.035). El análisis temporal de la incidencia reveló tres períodos de interés: 2007-2009, 2009-2013 y 2013-2023, con los respectivos cambios porcentuales anuales (*annual percent change* - APC) e intervalos de confianza del 95% (IC95%): (i) 91,4% (IC95% 24,9; 203,8; p-valor 0,002); (ii) -38,4% (IC95% -54,5; -26,8; p-valor 0,002); y (iii) 9,4% (IC95% 2,8; 21,1; p-valor 0,007). La mortalidad también mostró tres períodos distintos: una disminución entre 2007 y 2014 (APC -13,7%; IC95% -38,5; -2,8; p-valor 0,020), un aumento entre 2014 y 2019 (APC 27,6%; IC95% 8,5; 88,8; p-valor 0,010) y una nueva reducción entre 2019 y 2023 (APC -20,6%; IC95% -58,8; -6,4; p-valor 0,010). Se identificaron áreas calientes de incidencia en Fortaleza, Várzea Alegre y Pacoti.

**Conclusión:**

La leptospirosis en Ceará está asociada con lluvias intensas y condiciones socioambientales precarias. Medidas integradas de prevención, como la mejora del saneamiento y el control de roedores, son esenciales para reducir la carga de la enfermedad en el estado.

**Ethical aspects**

This research used public domain anonymized databases.

## Introduction 

Leptospirosis is a zoonosis transmitted between animals and humans, caused by spirochete-type bacteria belonging to the genus *Leptospira.* The disease constitutes a significant public health problem in tropical nations with developing economies, primarily due to high population densities, inadequate sanitation infrastructure, and rodent infestations, which serve as the main reservoirs of the disease ^1^. 

The dynamics of the disease are strongly associated with behavioral and socio-environmental factors, with a higher prevalence in areas characterized by high social inequality. In addition to this relationship with poverty, some authors highlight a probable lack of interest in addressing the problem, reinforced by the need for permanent or long-term treatment among those affected, which is costly. Consequently, in this context, leptospirosis came to be considered a neglected tropical disease ^2^.

A systematic review of the literature on leptospirosis studies in the Americas identified Brazil as one of the countries with the highest prevalence in South America. In the country, between 2010 and 2023, more than 45,000 cases of the disease were recorded, corresponding to an annual average of 3,361 cases. The mean case fatality ratio during this period was 9.0% ^3^. Specifically regarding incidence, data indicate that the Northeast region ranks third among the five Brazilian regions in terms of the absolute number of cases ^4^.

Nevertheless, the region is noteworthy for the lack of more detailed data on the disease's behavior in its states. National-level studies can be found in the literature ^5^ that address the temporal and spatial patterns of leptospirosis. However, few studies assess the incidence patterns of the disease in specific states of the Brazilian Northeast, such as Ceará.

Given this scenario, it is essential to investigate the incidence and mortality of leptospirosis in this state, taking into account its geographical and temporal characteristics. Ceará has high socio-environmental vulnerability, characterized by periods of intense and concentrated rainfall that cause river overflows, flooding in urban areas, and inundation in rural areas ^6^, in addition to poor sanitation conditions that favor the spread of the disease. According to data from epidemiological bulletins, between 2018 and 2023, the state recorded an annual average of 75 cases, with a case fatality rate of 24.5% in 2018. This rate positioned Ceará as the state with the third-highest leptospirosis incidence in the Northeast region ^7^.

Therefore, as a zoonosis and neglected tropical disease, leptospirosis has a significant impact on public health and presents considerable morbidity and mortality rates, which demand continuous monitoring. The application of spatial and temporal analysis methods to disease indicators is suitable for this study, as such analyses enable the identification of areas of greater epidemiological relevance in both space and time, thus favoring more precise and effective interventions. Accordingly, the objective of this research is to analyze the spatio-temporal pattern of leptospirosis incidence and mortality in the state of Ceará.

## Methods 

Study design and period

This was a descriptive observational study, with temporal, spatial, and spatio-temporal analysis. Data extraction was conducted between August and October 2024. Secondary publicly available data on leptospirosis notifications and deaths in Ceará were used. The disease is cataloged in the International Statistical Classification of Diseases and Related Health Problems 10th Revision (ICD-10) under code A27 ^8^. The study period covered data from 2007 to 2023. It should be noted that, at the time of extraction, data prior to 2007 were not available, and data after 2023 were still preliminary and therefore excluded. The study followed the Strengthening the Reporting of Observational Studies in Epidemiology (STROBE) recommendations ^9^.

Setting

All 184 municipalities of Ceará were used as the unit of analysis. The state is located in the Northeast region of Brazil. According to the Brazilian Institute of Geography and Statistics (*Instituto Brasileiro de Geografia e Estatística -* IBGE), in 2022, Ceará (Supplementary Figure 1) had an estimated population of over 8.7 million inhabitants and a territorial area of more than 148,000 km² ^8^.

Variables

Incidence and mortality rates were calculated using data on leptospirosis cases and deaths reported by municipalities across Ceará. In the temporal analysis, annual disease incidence and mortality were obtained by dividing the number of cases reported each year by the estimated population of the corresponding period. The result was multiplied by 100,000 inhabitants. 

Data sources

This study used secondary data freely available from the online portal of the Department of Informatics of the Brazilian National Health System (*Departamento de Informática do Sistema Único de Saúde* - DATASUS). Data were accessed via the Tabnet application, which allows access to notifications by municipality from the Notifiable Health Conditions Information System, and to deaths from the Mortality Information System ^8^. Data on the resident population were also obtained from DATASUS, based on population estimates produced by the IBGE (http://tabnet.datasus.gov.br/cgi/deftohtm.exe?ibge/cnv/popsvs2024br.def) ^8^.

Bias

Rates were age-adjusted in order to eliminate the effect of variations in the age distribution of the events analyzed. The population estimated by the 2010 Census, available on the IBGE website (https://www.ibge.gov.br/), was adopted as the study standard population. The use of a standard population is a crucial tool, as it enables more accurate comparisons and statistical analyses across diverse contexts. Nevertheless, the study is subject to the inherent limitations of using secondary data, such as underreporting, inconsistencies in records, and fluctuations in the coverage and quality of information systems over time.

Study size

No sampling techniques or geographic exclusions were applied in this study.

Statistical methods

For the temporal trend analysis, Joinpoint software was used. This software performs regression analysis based on inflection points, identifying the existence of one or more linear segments that indicate changes in the temporal trend of the phenomenon under study ^10^. The results of the temporal analysis were used to estimate the annual percent change (APC), along with its 95% confidence interval (95%CI), and to assess statistical significance (p-value<0.05). A positive and statistically significant APC indicates an increasing trend in the event being studied. A negative and significant APC indicates a decreasing trend. Nonsignificant APC values indicate a stationary trend. At the end of the period, it was possible to calculate the average annual percent change (AAPC), which demonstrates the change that occurred during the study period ^10^. 

For the spatial analysis, mean municipal incidence and mortality rates were calculated for each municipality of Ceará. They were standardized using the indirect method, with 2015 as the reference year. Both unadjusted rates are presented in a thematic map. Due to heterogeneity of rates and instability of values among neighboring municipalities, the data were smoothed using the local empirical Bayesian method ^11^. This method generates rates closer to reality by considering not only the value of a given municipality, but also its relation to its neighbors through a spatial proximity matrix ^11^. 

A binary contiguity matrix was also used, in which a value of 1 indicates the existence of a neighborhood between spatial units, and 0 indicates its absence. For the definition of neighborhood, the queen contiguity criterion was adopted, which considers as neighbors those units that share at least one vertex or edge ^12^. 

In addition, the Getis-Ord Gi* technique was applied. This analysis generates Z-scores for each municipality based on the selected indicator (incidence/mortality). Scores above the mean indicate clusters of high incidence (hotspots), while scores below the mean indicate areas of low incidence surrounded by similar areas (coldspots).

Finally, a spatio-temporal scan analysis was conducted to identify areas at higher risk of incidence and mortality. This analysis enables the more accurate identification of patterns that change over time and across locations. The Poisson model was adopted, considering the following criteria: (i) maximum cluster size equal to 50% of the exposed population; (ii) circular clusters; and (iii) 999 replications. The relative risk (RR) was calculated for each municipality; values greater than 1 indicate a risk higher than the state average ^10^.

Temporal regression by inflection points was conducted with version 5.0 of Joinpoint. Unadjusted rate calculations, local empirical Bayesian smoothing, and the Getis-Ord Gi* technique were performed using GeoDa version 1.22. The spatio-temporal scan technique was performed using SaTScan version 10.2. All maps were produced using QGIS version 3.16.

## Results 

Between 2007 and 2023, the Brazilian state Ceará recorded 1,319 cases of leptospirosis. Regarding the sociodemographic characteristics of the population, the highest proportion of cases was observed among men (83.9%), individuals aged 15-39 years (54.4%), Brown (Brazilian mixed race) (78.5%), and individuals with incomplete elementary education (28.7%). Complete recovery was the primary outcome (82.9%) of these cases. Of the total, 20.5% were work-related cases (Table 1).

Temporal analysis of incidence revealed three main periods of leptospirosis cases in the state (Figure 1A). In the first segment (2007-2009), there was a significant increase of 91.4% per year, with a 95% confidence interval (95%CI) of 24.9; 203.8 (p-value 0.002). In the second segment (2009-2013), there was a significant decrease of 38.4% per year (95%CI -54.5; -26.8; p-value 0.002), while the third segment (2013-2023) showed a new increase of 9.4% per year (95%CI 2.8; 21.1; p-value 0.007). The average annual percent change (AAPC) for the period was not significant.

The analysis of temporal trends in mortality also showed three segments (Figure 1B). Between 2007 and 2014, there was a significant reduction of 13.7% (95%CI -38.5; -2.8; p-value 0.020). This period was followed by a significant increase between 2014 and 2019, with a 27.6% (95%CI 8.5-88.8; p-value 0.010) rise. The last segment, 2019-2023, showed a decreasing trend, with mortality reduced by 20.6% per year (95%CI -58.8; -6.4; p-value 0.010). 

Spatial analysis of incidence showed that 37.5% of the municipalities in the state reported at least one case of leptospirosis during the period (Figure 2A). Fortaleza (70.8%), Várzea Alegre (9.0%), and Pacoti (6.5%), together, accounted for more than 85.0% of this total. After smoothing the data using the local empirical Bayesian method (Figure 2B), a more homogeneous distribution of incidence was observed, highlighting some additional municipalities beyond those already mentioned: Guaramiranga, Pereiro, Ereré, and Granjeiro.

Using the Getis-Ord Gi* method (Figure 2C), two regions with high-incidence hotspots were identified in the state. The first was located between the Maciço de Baturité and Sertão do Cariri regions, including the municipalities of Caridade, Palmácia, Guaramiranga, Baturité, and Redenção. The second was located in Vale do Jaguaribe, comprising the municipalities of Pereiro, Iracema, and Potiretama. Additionally, a hotspot was observed in Granjeiro, located in the Cariri region. The scan analysis (Figure 2D) identified municipalities with the highest risk of leptospirosis incidence across space and time. Pacoti, Ereré, and Várzea Alegre were particularly noteworthy. In total, 166 municipalities presented RR<1.

**Table 1:** Sociodemographic and clinical profile of reported leptospirosis cases. Fortaleza, 2025 (n=1,319)

Sociodemographic and clinic variables	n (%)
Sex	
Male	1,107 (83.9)
Female	212 (16.1)
Age group (years)	
Unknown	1 (0.1)
<1-14	101 (7.7)
15-39	718 (54.4)
40-64	408 (30.9)
65-79	82 (6.2)
>80	9 (0.7)
>Race/skin color	
Unknown	88 (6.7)
White	138 (10.5)
Black	43 (3.3)
Asian	14 (1.1)
Brown	1,035 (78.5)
Indigenous	1 (0.1)
Education level^a^	
Unknown	580 (44.0)
Illiterate	43 (3.3)
Incomplete elementary school	379 (28.7)
Complete elementary school	70 (5.3)
Incomplete high school	66 (5.0)
Complete high school	121 (9.2)
Incomplete higher education	14 (1.1)
Complete higher education	18 (1.4)
Work-related disease	
Unknown	420 (31.8)
Yes	270 (20.5)
No	629 (47.7)
Outcome^b^	
Unknown	81 (6.1)
Cure	1,093 (82.9)
Death	119 (9.0)

a 28 filled in as "Not applicable"; ^b^26 deaths from other causes.

**Figure 1:**
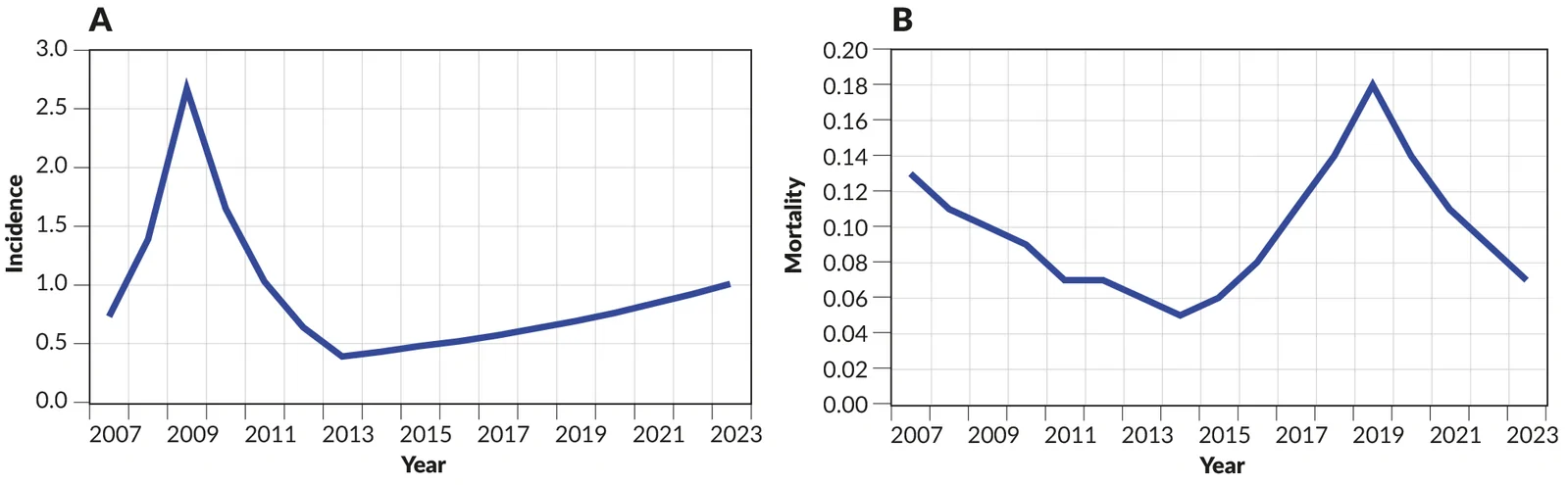
Annual (A) incidence and (B) mortality rates of leptospirosis. Ceará, 2007-2023 (n=1,319 for incidence; n=143 for mortality)

Regarding mortality, spatial analysis revealed that 17.5% of the municipalities in the state reported at least one leptospirosis-related death (Figure 3A), with Fortaleza (66.4%) and Caucaia (5.6%) being the most affected. After smoothing (Figure 3B), a more homogeneous distribution of mortality was observed, highlighting large areas including Fortaleza (the capital) and its metropolitan region, as well as other areas corresponding to Serra da Ibiapaba (western Ceará) and Vale do Jaguaribe (eastern Ceará). 

The Getis-Ord Gi* technique (Figure 3C) identified the main areas of mortality hotspots. The largest cluster corresponded to municipalities between Sertão do Canindé (Canindé, Paramoti, and Caridade), the West Coast (General Sampaio, Apuiarés, and Tejuçuoca), and Maciço de Baturité (Aratuba, Capistrano, and Mulungu). Other isolated hotspots were found in the municipalities of Pereiro, Moraújo, Pacujá, Maranguape, Palmácia, and Chorozinho. The scan analysis for relative risk (Figure 3D) identified seven municipalities with RR>1 in the spatio-temporal assessment, namely: Fortaleza, Palmácia, Aratuba, Tururu, General Sampaio, Moraújo, and Graça. The other 154 municipalities in the state presented RR<1.

Finally, spatio-temporal clusters of incidence and mortality were identified in the state. For incidence (Figure 4A), Icó was the central cluster in Ceará, with a RR 27.2 during 2008-2009 (p-value<0.001). Other significant clusters were identified in Guaramiranga, between 2007 and 2014 (RR 66.2; p-value<0.001), Fortaleza, between 2007 and 2012 (RR 3.1; p-value<0.001), and Várzea Alegre, between 2016 and 2022 (RR 2.3). 

The analysis also identified four additional clusters during the period, but they were not statistically significant, namely: Várzea Alegre in 2011 (RR 7.8; p-value 0.078), Farias Brito during 2018-2022 (RR 5.6; p-value 0.340), Independência in 2012 (RR 17.8; p-value 0.711), and Sobral in 2022 (RR 5.1; p-value 0.790). For mortality (Figure 4B), only two observations were found, both of which were not statistically significant: Caucaia from 2016 to 2022 (RR 5.1; p-value 6.704) and Paracuru from 2007 to 2011 (RR 2.2; p-value 0.852). All municipalities described in the results are listed in Supplementary Figure 2.

**Figure 2:**
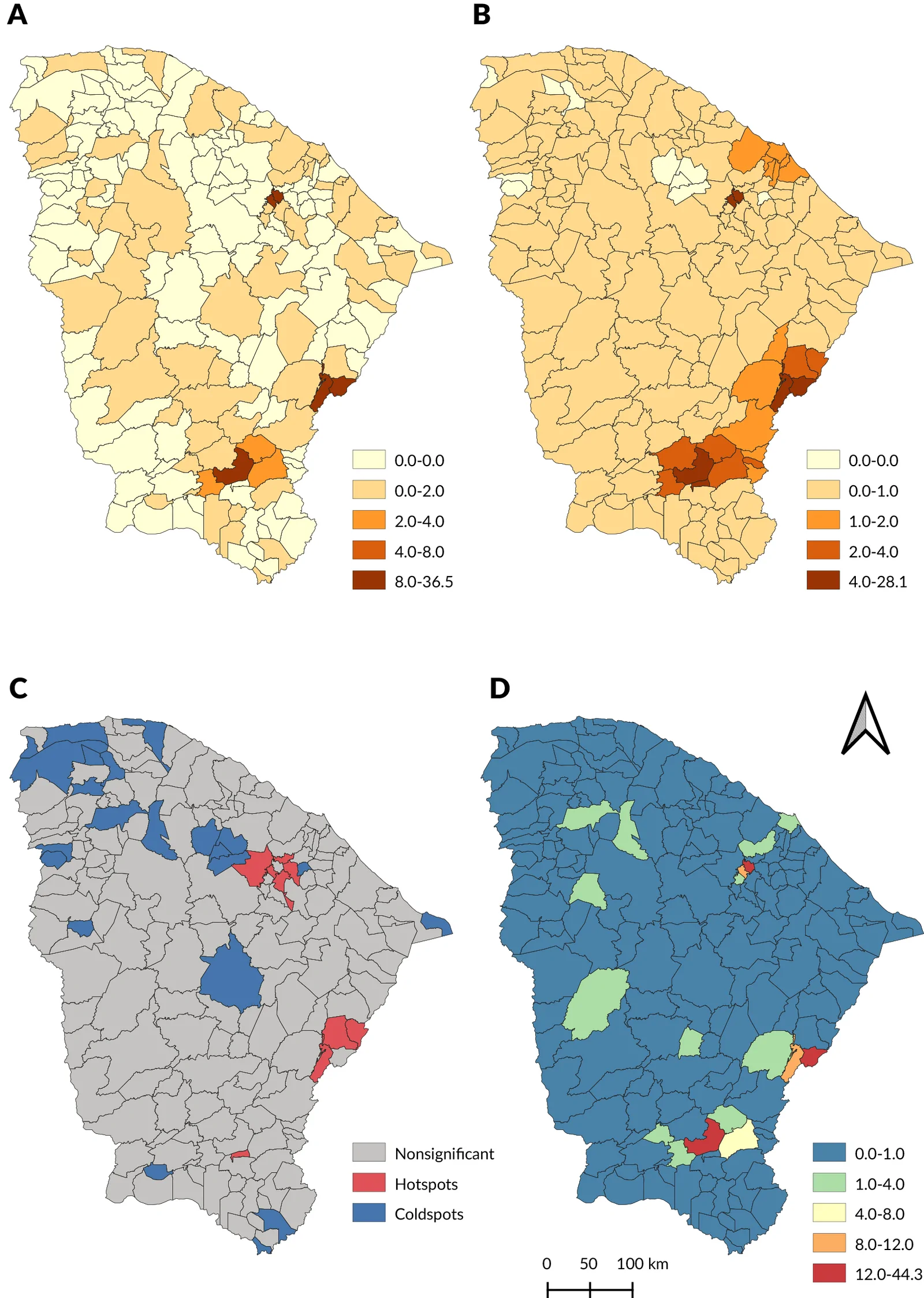
Annual spatial distribution of leptospirosis incidence: (A) crude rate analysis, (B) after local empirical Bayesian smoothing, (C) by Getis-Ord Gi* and (D) relative risk (RR). Ceará, 2007-2023 (n=1,319)

**Figure 3:**
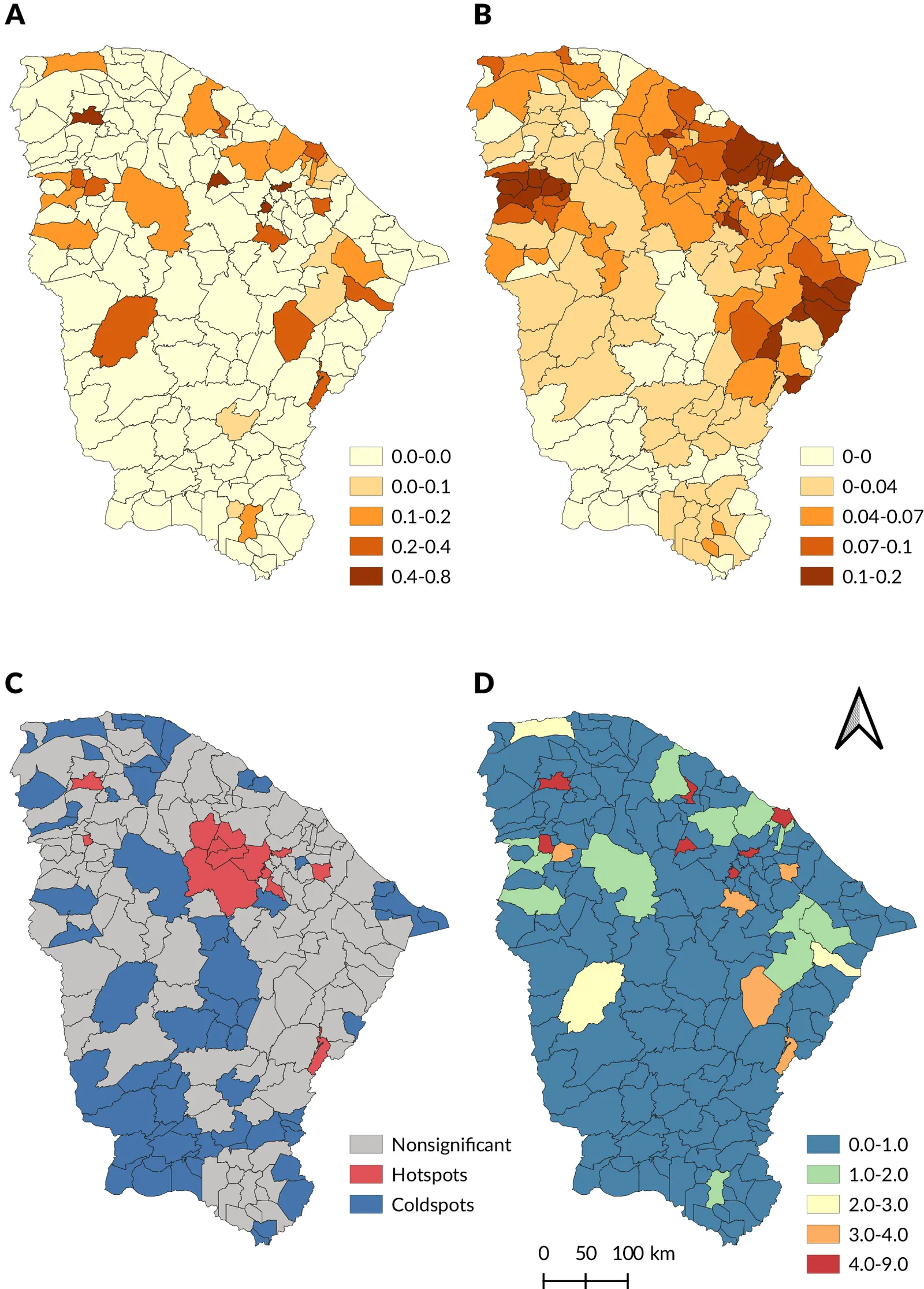
Annual spatial distribution of leptospirosis mortality: (A) crude rate analysis, (B) after local empirical Bayesian smoothing, (C) by Getis-Ord Gi* and (D) relative risk (RR). Ceará, 2007-2023 (n=143)

## Discussion 

The profile of leptospirosis cases identified in Ceará reflects a pattern already recognized in different contexts, both in Brazil and in other countries, reinforcing that certain population groups are systematically more exposed to the disease. The significant concentration of cases among adult men, with low educational attainment and identified as Brown or White, highlights the relationship between social, economic, and occupational factors and exposure to leptospirosis ^2^^,^^13^^-^^15^. 

These individuals are often engaged in occupations that require contact with unhealthy environments or exposure to flood-affected areas, such as agricultural workers, veterinarians, hunters, travelers, and military personnel ^16^. Thus, this is a disease marked by structural inequalities, in which occupation, education, and racial or ethnic belonging shape the level of exposure and risk of illness from leptospirosis. 

The finding on the temporal behavior of incidence is consistent with patterns reported in studies from Santa Catarina (Southern Brazil), which, similarly to this study, applied time series analysis using secondary data. Although the climatic context of that state differs from Ceará, with more continuous rainfall throughout the year ^17^, these findings underscore the central role of rainfall in the disease's dynamics, in line with the specialized literature ^12^^,^^18^^,^^19^.

Few studies have examined the spatial distribution of the disease incidence in Ceará. However, the municipalities in need of greater attention are not considered large urban centers. For example, Pacoti, Pereiro, and Várzea Alegre reported a high incidence after Bayesian smoothing and a risk above the state average, which may be associated with a history of an outbreak that occurred approximately ten years ago ^20^^,^^21^. 

Spatial analysis of incidence with and without Bayesian smoothing highlights relevant differences in the identification of priority municipalities. In the cases of Pacoti, Pereiro, and Várzea Alegre, smoothing not only maintained the high incidence previously identified but also emphasized elevated risk in adjacent areas. This pattern may be associated with the small population size of these municipalities, which makes their rates more susceptible to fluctuations due to the small number of cases. Moreover, rural areas also tend to exhibit higher leptospirosis occurrence due to greater exposure to areas with signs of rodent activity, animal husbandry, and grain storage sites, as well as proximity to rivers and streams ^22^.

Urban areas, in turn, involve other types of exposure, such as contact with septic tanks, proximity to vacant lots, garbage accumulation, direct contact with rodents, inadequate sources of drinking water, and flooding ^14^^,^^23^. The identification of incidence clusters also revealed predominance in smaller municipalities.

However, the finding that Fortaleza, the capital and the most populous city in the state, and Sobral, the fifth most populous, were among the main clusters is consistent with current research. These highlight large urban centers as the most significant contributors to the overall number of leptospirosis cases. In these areas, urban vulnerabilities such as improper solid waste disposal, failures in waste collection, and contamination of water resources significantly contribute to the environmental spread of the bacteria. These factors are intensified during periods of heavy rainfall, favoring an increase in cases. A study conducted in Fortaleza identified a significant increase in the incidence of diseases associated with inadequate waste management, including leptospirosis, reinforcing the relevance of this urban context in the transmission of the disease ^24^.

**Figure 4:**
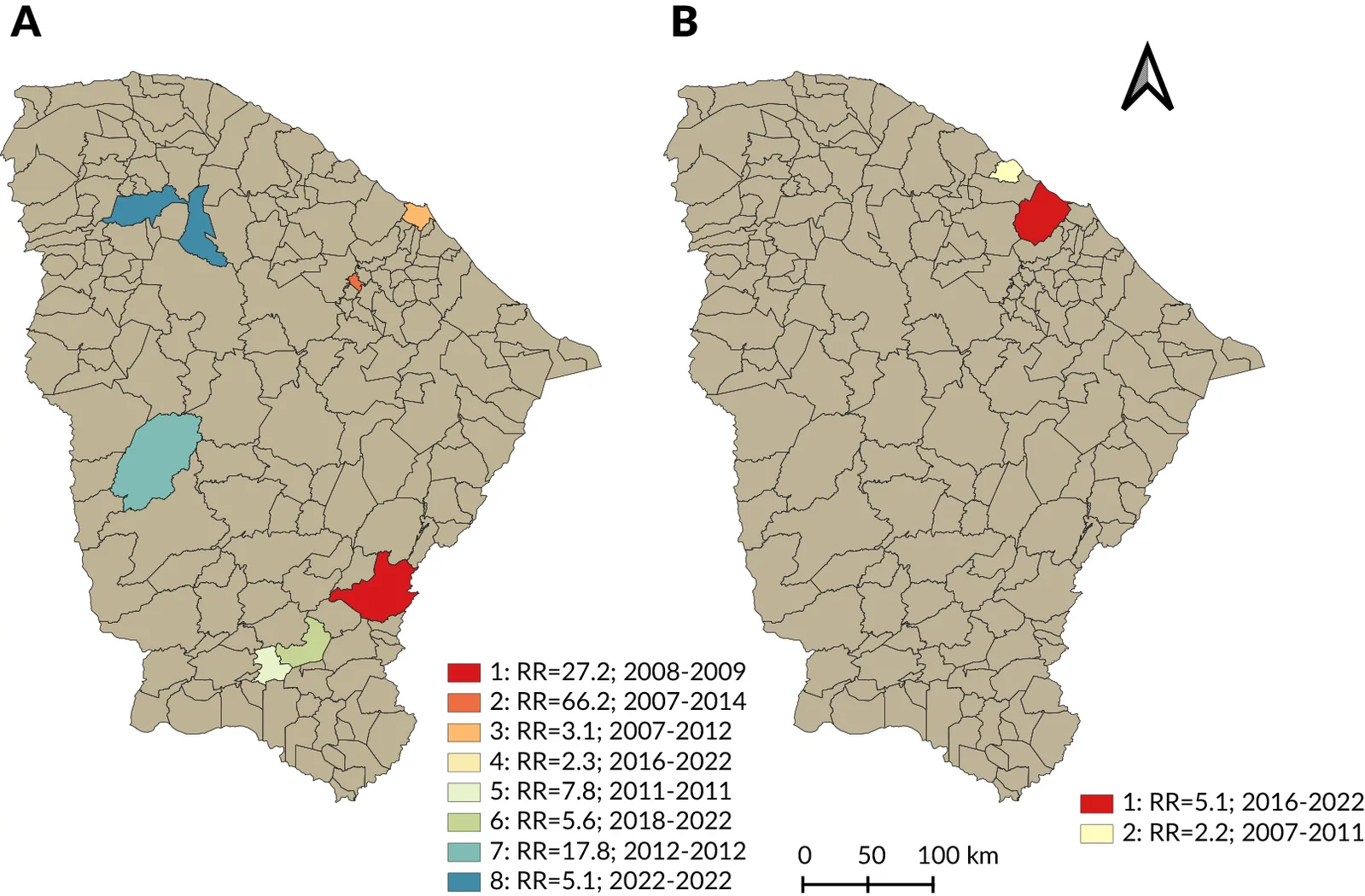
Annual identification of clusters, periods, and relative risk (RR): (A) leptospirosis incidence and (B) mortality. Ceará, 2007-2023 (n=1,319 for incidence; n=143 for mortality)

Regarding mortality, its temporal evolution has been little discussed in the literature of Ceará. Although studies indicate a decreasing trend in deaths from the disease at the national and international levels ^2^^,^^25^^-^^27^, this study reveals a more complex dynamic in Ceará, with distinct periods of increase and decline in mortality. Furthermore, the mortality findings reinforce patterns already observed for incidence, with higher occurrence among men, individuals with low educational attainment, and populations living in vulnerable contexts. Environmental factors, such as high rainfall and flooding, are also significantly associated with leptospirosis-related deaths, contributing to the severity of the outcomes ^2^^,^^14^^,^^25^^-^^27^.

Spatial analysis highlighted Fortaleza as the municipality with the highest mortality risk in the state and Caucaia as the central cluster. It is noteworthy that no other studies in the literature have evaluated the spatial distribution of the disease in Ceará, making comparisons impossible. Nonetheless, national studies conducted in other states, such as Rio Grande do Sul between 2007 and 2019, also identified concentrations of deaths and higher risk in capitals and metropolitan regions. Clusters of high incidence and risk were identified in more densely populated urban areas, corroborating the findings of the present study, which highlights the association between urban vulnerabilities and higher leptospirosis mortality ^28^.

In addition to rainfall, other studies evaluating the spatial dynamics of leptospirosis mortality reinforce the influence of socioeconomic vulnerability ^29^. However, some of them point out ^5^ that causal determinants are better identified in incidence than in mortality. Thus, studies on leptospirosis should consider social, environmental, and healthcare access inequalities as contributing factors to illness ^30^, as these deficiencies hinder disease control and reinforce its neglect.

This study revealed variations in leptospirosis incidence and mortality over the analyzed period, with alternating phases of increase and reduction, without evidence of a sustained general downward trend. On the one hand, the concentration of cases in inland municipalities, such as Pacoti, Pereiro, and Várzea Alegre, reflects the influence of environmental and socioeconomic conditions in areas not considered urban centers. On the other hand, the highest mortality rates are recorded in the capital, Fortaleza, and in Caucaia, a municipality in its metropolitan region, highlighting the fragility of densely populated areas, which is exacerbated by episodes of heavy rainfall. 

Therefore, the temporal dynamics indicate the influence of environmental and seasonal factors. At the same time, the spatial distribution reveals that risk is not limited to urban centers, but also affects inland municipalities, which are marked by persistent vulnerabilities. Some limitations of this study should be noted, particularly those related to the use of secondary databases, which are subject to incompleteness or non-adherence by health professionals in completing disease notification forms. Therefore, the findings are sensitive to ecological fallacy. Furthermore, population data may not fully reflect reality, meaning that the results should be interpreted as estimates. It is also important to emphasize that the use of Bayesian rates may cause spatial dependence in the data, as the method's characteristics rely on maximum likelihood; thus, results should be interpreted with caution.

In conclusion, leptospirosis in Ceará exhibits a heterogeneous spatio-temporal pattern, influenced by social, environmental, and structural factors. These findings underscore the importance of implementing integrated prevention and control measures, including the expansion of sanitation infrastructure, monitoring of high-risk areas, rodent control, and health education initiatives. Strengthening epidemiological surveillance and prioritizing public policies in the most critical regions are crucial to reducing the burden of leptospirosis and protecting the most vulnerable populations.

## Data Availability

The data utilized in the research were deposited in the Open Science Framework, available at: https://osf.io/h7s8z/?view_only=89a03da727e84108a642b5a3e64c54ef.
