# Supplementary material for: Leptospirosis incidence and mortality: a spatio-temporal analysis, Ceará, 2007-2023
Source: Epidemiol Serv Saude. 2026 May 10;35:e20250108. doi: 10.1590/S2237-96222026v35e20250108.en (PMC13686361; doi:10.1590/S2237-96222026v35e20250108.en)
Supplement: Supplementary file 1 [file 2237-9622-ress-35-e20250108-supl1.pdf]

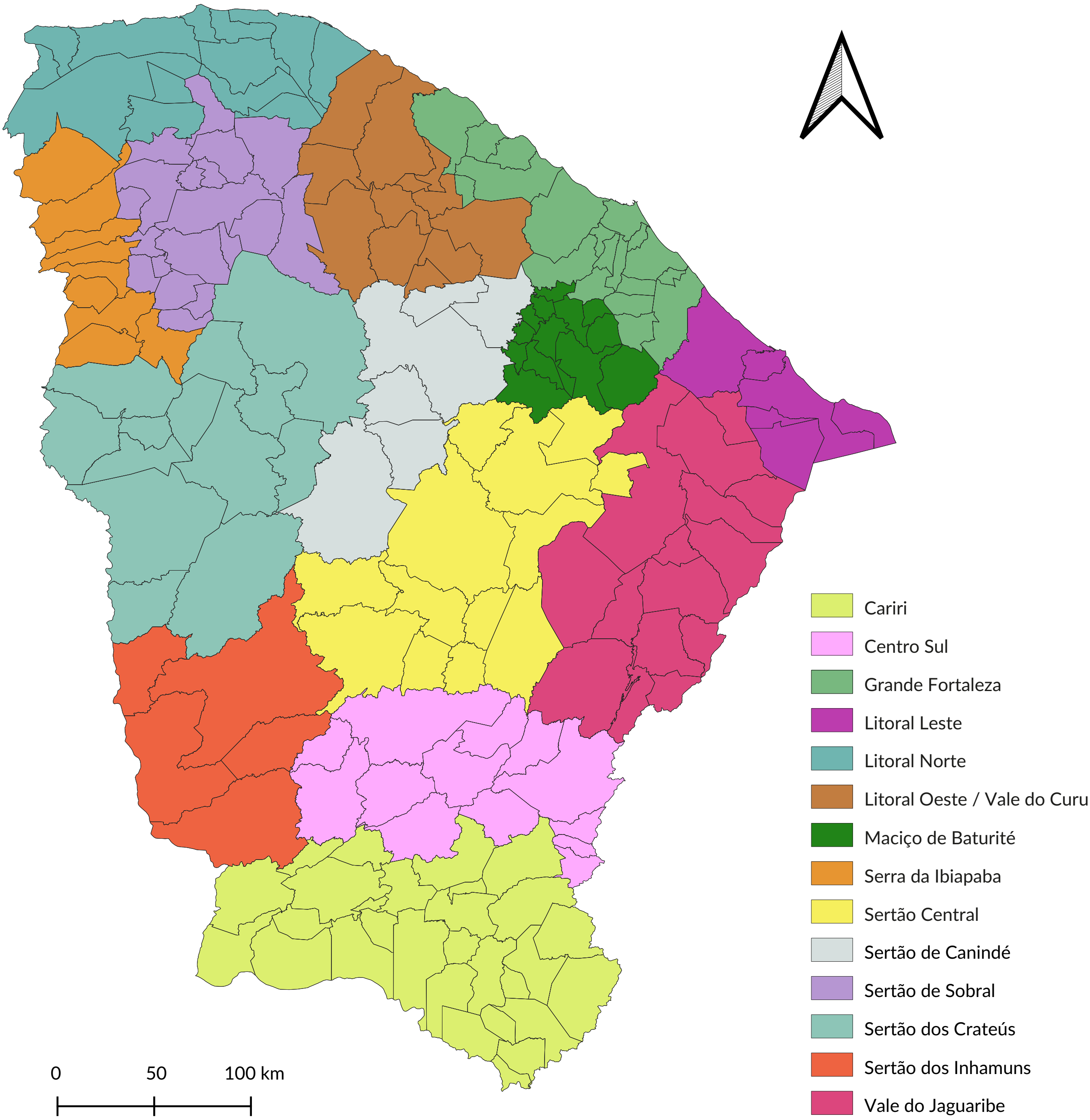

Supplementary figure 1. State of Ceará by planning regions. Ceará, 2025

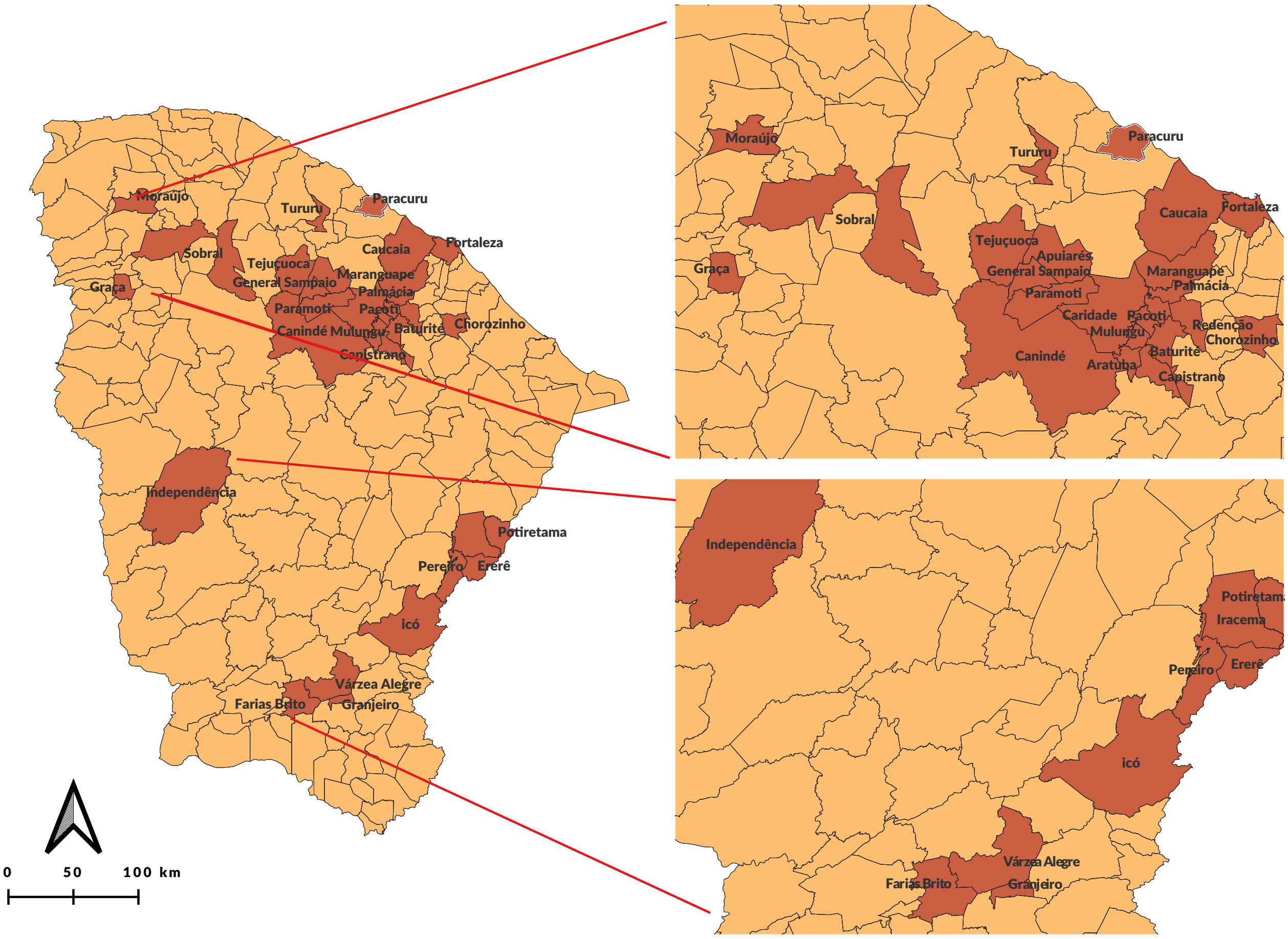

Supplementary figure 2. Municipalities of interest regarding leptospirosis incidence and mortality findings. Ceará, 2007–2023 (n=1,319 for incidence; n=143 for mortality)
