# Supplementary material for: Leptospirosis incidence and mortality: a spatio-temporal analysis, Ceará, 2007-2023
Source: Epidemiol Serv Saude. 2026 May 10;35:e20250108. doi: 10.1590/S2237-96222026v35e20250108.en (PMC13686361; doi:10.1590/S2237-96222026v35e20250108.en)
Supplement: Supplementary file 2 [file 2237-9622-ress-35-e20250108-supl2.pdf]

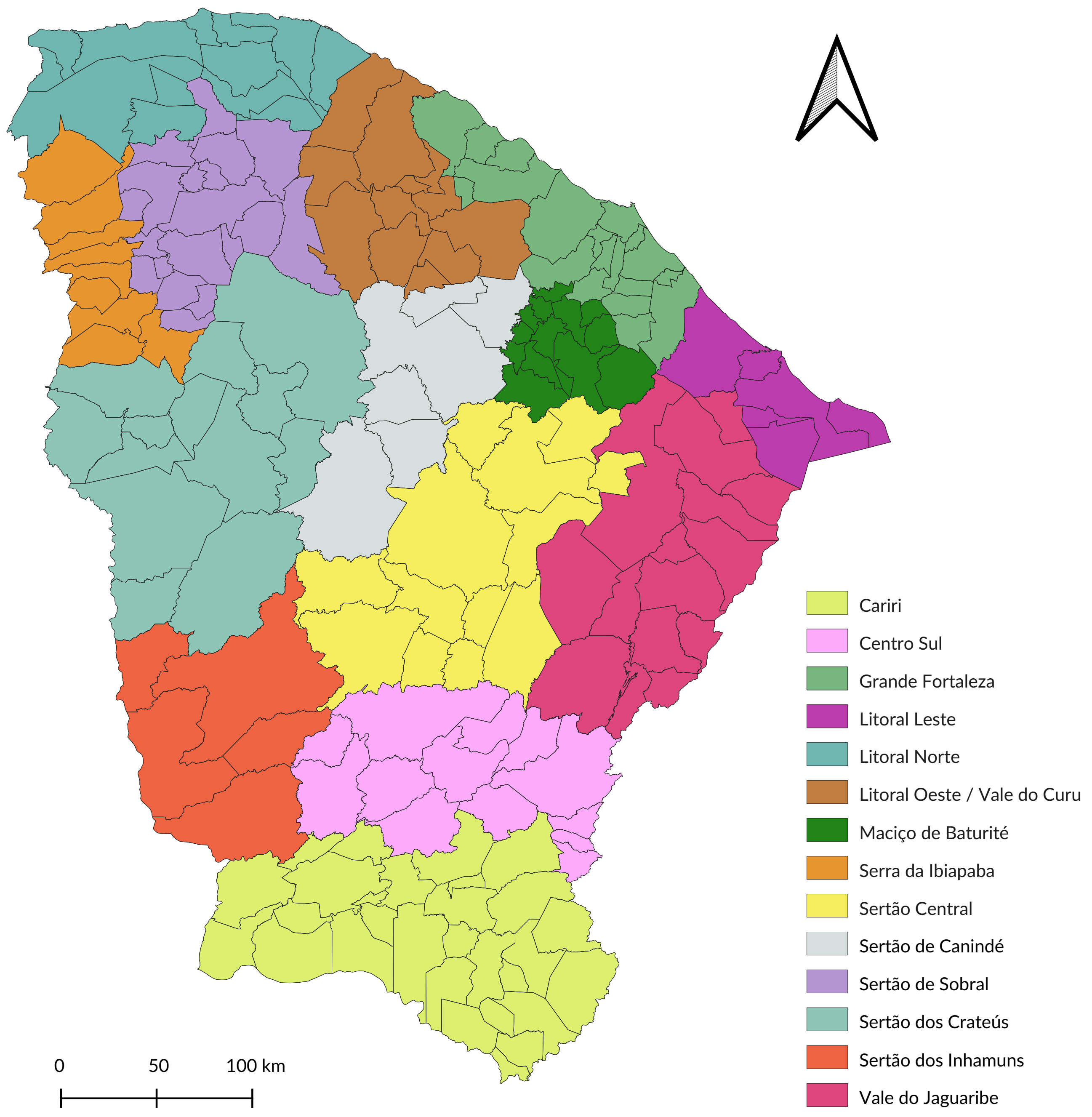

Figura Suplementar 1. Estado do Ceará por regiões de planejamento. Ceará, 2025

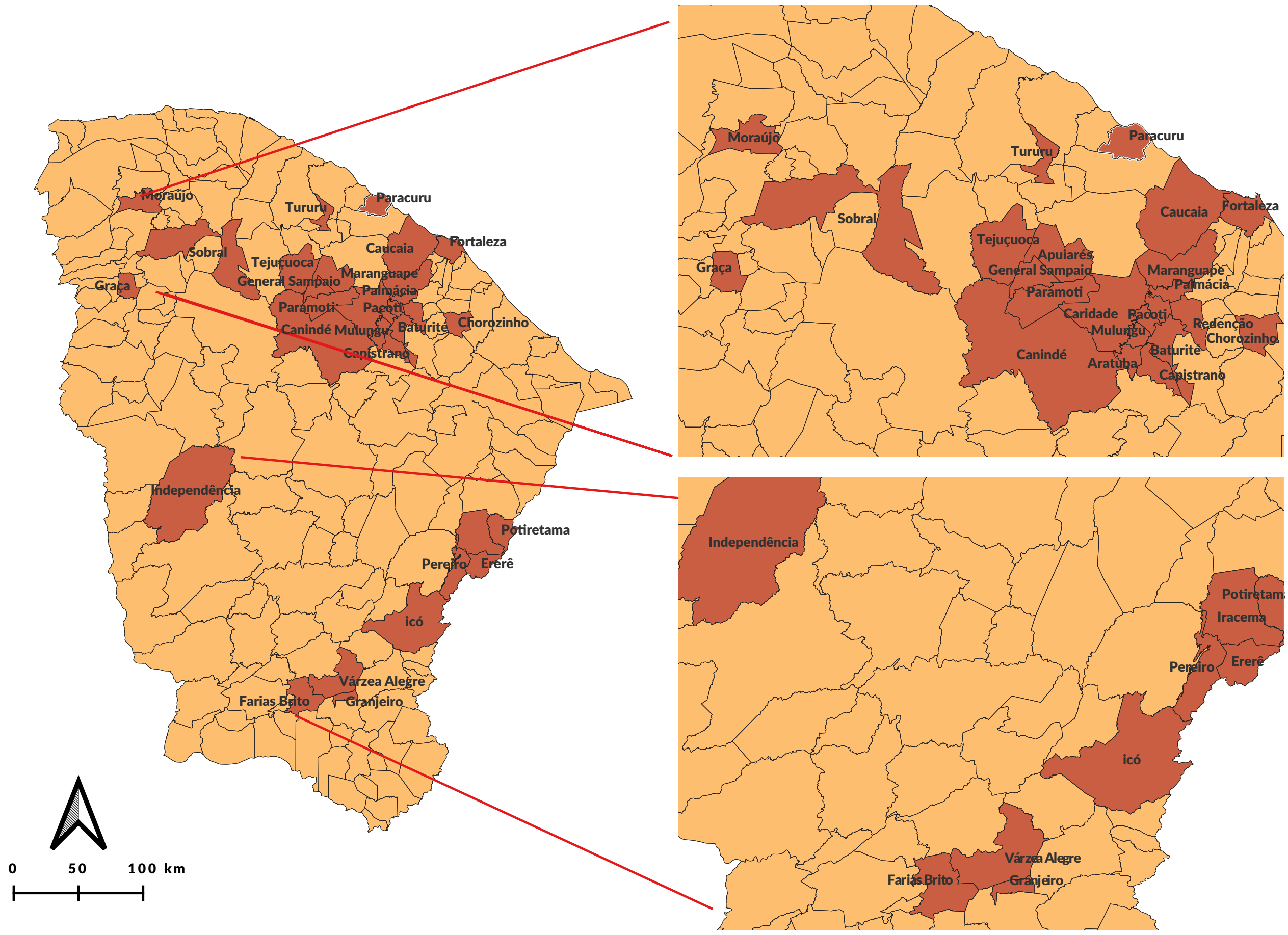

Figura Suplementar 2. Municípios de interesse quanto aos achados de incidência e de mortalidade por leptospirose. Ceará, 2007-2023 (n=1.319 para incidência; n=143 para mortalidade)
