# Supplementary material for: Leptospirosis incidence and mortality: a spatio-temporal analysis, Ceará, 2007-2023
Source: Epidemiol Serv Saude. 2026 May 10;35:e20250108. doi: 10.1590/S2237-96222026v35e20250108.en (PMC13686361; doi:10.1590/S2237-96222026v35e20250108.en)
Supplement: Translation

# Translation: Incidência e mortalidade por leptospirose: análise espaço-temporal, Ceará, 2007-2023

**DOI:** 10.1590/S2237-96222026v35e20250108.pt

**Authors:** Isabella Martins Camelo, Bruno Victor Barros Cabral, Davi Gomes Sousa, George Jó Bezerra Sousa, Maria Lúcia Duarte Pereira

**Affiliations:** ¹ Universidade Estadual do Ceará, Centro de Ciências da Saúde, Fortaleza, CE, Brazil Universidade Estadual do Ceará Centro de Ciências da Saúde Fortaleza CE Brazil; ² Ministério da Saúde, Brasília, DF, Brazil Ministério da Saúde Brasília DF Brazil

**Keywords:** Análise Espacial, Doença de Weil, Epidemiologia, Leptospirose, Análise Espaço-Temporal

## Abstract

**Objetivo:**

Analisar o padrão espaço-temporal da incidência e da mortalidade por leptospirose no Ceará entre 2007 e 2023.

**Métodos:**

Estudo observacional descritivo, com análise temporal (regressão por pontos de inflexão), espacial (suavização bayesiana e Getis-Ord Gi*) e espaço-temporal (varredura) para identificar áreas de risco, com dados secundários do Departamento de Informática do Sistema Único de Saúde a partir da análise de taxas de incidência e de mortalidade por leptospirose no Ceará entre 2007 e 2023.

**Resultados:**

O estudo registrou 1.319 casos de leptospirose, com maioria de homens (83,9%, n=1.107) jovens (15-39 anos) e pardos (78,5%, n=1.035). A análise temporal da incidência evidenciou três períodos de interesse: 2007-2009, 2009-2013 e 2013-2023, com as respectivas variações percentuais anuais (*annual percent change* - APC) e os intervalos de confiança de 95% (IC95%) sendo: (i) 91,4% (IC95% 24,9; 203,8; p-valor 0,002); (ii) -38,4% (IC95% -54,5; -26,8; p-valor 0,002); e (iii) 9,4% (IC95% 2,8; 21,1; p-valor 0,007). A mortalidade apresentou três períodos distintos: redução entre 2007 e 2014 (APC -13,7%; IC95% -38,5; -2,8; p-valor 0,020), aumento entre 2014 e 2019 (APC 27,6%; IC95% 8,5; 88,8; p-valor 0,010) e nova queda entre 2019 e 2023 (APC -20,6%; IC95% -58,8; -6,4; p-valor 0,010). Áreas quentes de incidência foram identificadas em Fortaleza, Várzea Alegre e Pacoti.

**Conclusão:**

A leptospirose no Ceará está associada a chuvas intensas e a condições socioambientais precárias. Medidas integradas de prevenção, como a melhoria do saneamento e o controle de roedores, são essenciais para reduzir a doença no estado.

Aspectos éticos

Esta pesquisa utilizou bancos de dados de domínio público e anonimizados.

## Introdução 

A leptospirose é uma zoonose transmitida entre animais e humanos, provocada por bactérias do tipo espiroqueta, pertencentes ao gênero *Leptospira.* A doença é um problema significativo de saúde pública em nações tropicais com economias em desenvolvimento devido, principalmente, a altas densidades demográ­ficas, infraestrutura sanitária inadequada e infestações de roedores, principais reservatórios da doença ^1^. 

Observa-se que a dinâmica da doença está fortemente associada a fatores comportamentais e socioambientais, sendo mais prevalente em áreas marcadas pela elevada desigualdade social. Além de tal relação com a pobreza, autores evidenciam um provável desinteresse na resolução da problemática em torno da doença, corroborado pela necessidade de tratamentos permanentes ou de longo prazo por parte de quem a adquire, os quais são de alto custo. Logo, nesse contexto, a leptospirose passou a ser considerada uma doença tropical negligenciada ^2^.

Ao realizar uma revisão sistemática da literatura dos estudos de leptospirose nas Américas, identificou-se que o Brasil era um dos principais países quanto à sua prevalência na América do Sul. No país, entre 2010 e 2023, foram registrados mais de 45 mil casos da doença, o que corresponde a uma média anual de 3.361 casos. A taxa de letalidade média durante esse período foi 9,0% ^3^. Especificamente, em relação à incidência, levanta-se que a região Nordeste ocupa a terceira posição entre as cinco regiões do Brasil levando-se em conta o número absoluto de casos ^4^.

Entretanto, a região chama atenção quanto à ausência de dados mais aprofundados acerca do comportamento da doença nos seus estados integrantes. É possível encontrar na literatura estudos em nível nacional ^5^ que abordam padrões temporais e espaciais quanto à leptospirose. No entanto, há poucos estudos que avaliem os padrões de incidência da doença em estados específicos da região Nordeste do Brasil, como o Ceará.

Diante desse cenário, observa-se a necessidade de um estudo sobre a incidência e a mortalidade por leptospirose nesse estado, tendo em vista as suas características geográficas e temporais. O Ceará apresenta elevada vulnerabilidade socioambiental, marcada por períodos de chuvas intensas e concentradas que causam transbordamento de rios, alagamentos em áreas urbanas e inundações em áreas rurais ^6^, além de condições precárias de saneamento, o que favorece a disseminação da doença. Dados de boletim epidemiológico evidenciam que, entre 2018 e 2023, houve uma média de 75 casos por ano, com letalidade em 2018 de 24,5%. Esse índice posiciona o estado como o terceiro maior em incidência de leptospirose na região Nordeste ^7^.

Portanto, a leptospirose como zoonose e doença tropical negligenciada possui um impacto significativo na saúde pública e apresenta taxas consideráveis de morbidade e de mortalidade, o que exige monitoramento contínuo. Assim, a aplicação de métodos de análise espacial e temporal diante dos indicadores da doença adequa-se ao estudo, pois essas análises permitem a identificação de áreas que possuem maior relevância quanto à doença no espaço e no tempo, de modo a favorecer intervenções mais precisas e eficazes. Por conseguinte, esta pesquisa tem como objetivo analisar o padrão espaço-temporal da incidência e da mortalidade por leptospirose no estado do Ceará.

## Métodos 

Desenho e período do estudo

Tratou-se de estudo observacional descritivo, com análise temporal, espacial e espaço-temporal. A extração de dados ocorreu entre agosto e outubro de 2024. Utilizaram-se dados secundários disponíveis publicamente quanto às notificações e aos óbitos por leptospirose no Ceará. A doença está catalogada na 10ª revisão da Classificação Internacional de Doenças (CID-10) sob o código A27 ^8^. O período do estudo abrangeu dados entre 2007 e 2023. Salienta-se que, durante a extração, os dados anteriores a 2007 não estavam disponíveis, e os posteriores a 2023 apresentavam-se de forma preliminar, portanto foram excluídos da coleta. O estudo seguiu as recomendações do Strengthening the Reporting of Observational Studies in Epidemiology (STROBE) ^9^.

Contexto

Utilizaram-se todos os 184 municípios do Ceará como unidade de análise. O estado localiza-se na região Nordeste do Brasil. De acordo com o Instituto Brasileiro de Geografia e Estatística (IBGE), o Ceará (Figura Suplementar 1), em 2022, possuía uma população estimada de mais de 8,7 milhões de habitantes e extensão territorial de mais de 148 mil km² ^8^.

Variáveis

Para o cálculo da incidência e da mortalidade, foram utilizados dados de casos/óbitos de leptospirose por município de notificação em todo o Ceará. Para a análise temporal, a incidência e a mortalidade anual da doença foram calculadas utilizando-se o número de casos notificados em cada ano, dividido pela população estimada dos períodos coletados. O resultado foi multiplicado por 100 mil habitantes. 

Fonte de dados

Este estudo utilizou dados secundários obtidos livremente no portal eletrônico do Departamento de Informática do Sistema Único de Saúde (DATASUS). Os dados foram acessados pela aplicação Tabnet, que permite acesso às notificações por município do Sistema de Informação de Agravos de Notificação e, aos óbitos, pelo Sistema de Informação sobre Mortalidade ^8^. Os dados acerca da população residente também foram coletados no DATASUS a partir de estimativas populacionais realizadas pelo IBGE (http://tabnet.datasus.gov.br/cgi/deftohtm.exe?ibge/cnv/popsvs2024br.def) ^8^.

Viés

As taxas foram ajustadas por idade, com o objetivo de eliminar o efeito das variações na composição etária dos eventos analisados. Adotou-se como população padrão do estudo aquela estimada pelo Censo de 2010, disponível no site do IBGE (https://www.ibge.gov.br/). O uso da população padrão é uma ferramenta importante, pois permite comparações e análises estatísticas mais precisas entre diferentes contextos. Ainda assim, o estudo está sujeito às limitações inerentes ao uso de dados secundários, como subnotificações, inconsistências nos registros e oscilações na cobertura e na qualidade dos sistemas de informação ao longo do tempo.

Tamanho do estudo

Não foram utilizadas técnicas de amostragem ou exclusões geográficas neste estudo.

Métodos estatísticos

Para o cálculo da análise temporal, utilizou-se o software Joinpoint. Ele realiza a análise por meio da regressão baseada em pontos de inflexão, identificando a existência de um ou mais segmentos lineares, que indicam alguma mudança na tendência temporal do objeto analisado ^10^. Os resultados da análise temporal são utilizados para estimar a variação percentual anual (*annual percent change -* APC), além de determinar seu intervalo de confiança de 95% (IC95%) e avaliar a significância estatística (p-valor<0,05). Uma APC positiva e significativa indica uma tendência de aumento do fenômeno estudado. Uma APC negativa e significativa indica uma tendência decrescente. Valores de APC não significativos indicam uma tendência estacionária. Ao final do período, foi possível obter a variação percentual anual média (*average annual percent change -* AAPC), que demonstra como ocorreu a mudança durante o período estudado ^10^. 

Para a análise espacial, foram criadas as variáveis taxa de incidência municipal média e mortalidade municipal média para cada município do Ceará. Elas foram padronizadas pelo método indireto, utilizando como referência o ano médio do período (2015). Ambas as taxas brutas são apresentadas em um mapa temático. Devido à heterogeneidade das taxas e à instabilidade dos valores entre municípios vizinhos, os dados foram suavizados pelo método bayesiano empírico local ^11^. Esse método é necessário para gerar taxas mais próximas da realidade, pois considera não apenas o valor da taxa de um determinado município, mas também o considera em relação às suas fronteiras, através de uma matriz de proximidade espacial ^11^. 

Destaca-se também a utilização de uma matriz de proximidade binária, na qual o valor 1 indica a existência de vizinhança entre as unidades espaciais, e o valor 0 indica a ausência dessa relação. Para a definição de vizinhança, adotou-se o critério da rainha (*queen contiguity*), que considera como vizinhas as unidades que compartilham ao menos um vértice ou uma aresta ^12^. 

Além disso, foi aplicada a técnica Getis-Ord Gi*. Essa análise cria escores Z para cada município a partir do indicador desejado (incidência/mortalidade). Escores maiores que a média indicam áreas de alta incidência agrupadas (áreas quentes), e escores menores que a média indicam áreas de baixa incidência cercadas por áreas semelhantes (áreas frias).

Por fim, foi realizada uma análise de varredura espaço-temporal para identificar as áreas com maior risco de incidência e as áreas com maior risco para mortalidade. O uso dessa análise propicia uma melhor identificação de padrões que mudam ao longo do tempo e em diferentes locais. Para tal, foi adotado o modelo de Poisson, considerando-se os seguintes critérios: (i) tamanho máximo do aglomerado igual a 50% da população exposta; (ii) aglomerados circulares; e (iii) 999 replicações. O risco relativo (RR) foi calculado para cada município; valores >1 evidenciam um risco maior que a média do estado ^10^.

A regressão temporal por pontos de inflexão foi realizada através do software Joinpoint 5.0. O cálculo das taxas brutas, das bayesianas empíricas locais e da técnica de Getis-Ord Gi* utilizaram-se do software GeoDa 1.22. A técnica de varredura espaço-temporal foi realizada no software SaTScan 10.2. Todos os mapas foram elaborados utilizando o programa QGIS 3.16.

## Resultados 

Entre 2007 e 2023, o Ceará registrou 1.319 casos de leptospirose. Quanto às características sociodemográficas da população, a maior proporção de casos foi observada em homens (83,9%), entre 15 e 39 anos de idade (54,4%), pardos (78,5%) e com ensino fundamental incompleto (28,7%). A cura foi o principal desfecho (82,9%) desses casos. Do total, 20,5% eram casos re­lacionados às atividades laborais (Tabela 1).

A análise da evolução temporal da incidência evidenciou os três principais momentos dos casos de leptospirose no estado (Figura 1A). No primeiro segmento (2007-2009), houve crescimento sig­nificativo de 91,4% ao ano (IC95% 24,9; 203,8; p-valor 0,002). No segundo segmento (2009-2013), observou-se queda significativa dos casos, de 38,4% ao ano (IC95% -54,5; -26,8; p-valor 0,002), enquanto o terceiro segmento (2013-2023) evidenciou novo aumento de 9,4% ao ano (IC95% 2,8; 21,1; p-valor 0,007). A AAPC do período não foi significativa.

**Tabela 1:** Caracterização sociodemográfica e clínica dos casos notificados de leptospirose. Fortaleza, 2025 (n=1.319)

Variáveis sociodemográficas e clínicas	n (%)
Sexo	
Masculino	1.107 (83,9)
Feminino	212 (16,1)
Faixa etária (anos)	
Ignorado	1 (0,1)
<1-14	101 (7,7)
15-39	718 (54,4)
40-64	408 (30,9)
65-79	82 (6,2)
>80	9 (0,7)
Raça/cor da pele	
Ignorado	88 (6,7)
Branco	138 (10,5)
Preta	43 (3,3)
Amarela	14 (1,1)
Parda	1.035 (78,5)
Indígena	1 (0,1)
Escolaridade^a^	
Ignorado	580 (44,0)
Analfabeto	43 (3,3)
Fundamental incompleto	379 (28,7)
Fundamental completo	70 (5,3)
Médio incompleto	66 (5,0)
Médio completo	121 (9,2)
Superior incompleto	14 (1,1)
Superior completo	18 (1,4)
Doença relacionada ao trabalho	
Ignorado	420 (31,8)
Sim	270 (20,5)
Não	629 (47,7)
Desfecho^b^	
Ignorado	81 (6,1)
Cura	1.093 (82,9)
Óbito	119 (9,0)

a 28 preenchidos como "Não se aplica"; ^b^26 óbitos por outras causas.

**Figura 1:** Taxas anuais: (A) incidência e (B) mortalidade por leptospirose. Ceará, 2007-2023 (n=1.319 para incidência; n=143 para mortalidade)

O estudo acerca da evolução temporal da mortalidade também evidenciou três segmentos (Figura 1B). Houve, entre 2007 e 2014, uma redução significativa, de 13,7% (IC95% -38,5; -2,8; p-valor 0,020) da taxa. Esse período é seguido por um crescimento significativo, entre 2014 e 2019, de 27,6% (IC95% 8,5; 88,8; p-valor 0,010). O último segmento, 2019-2023, apresentou tendência decrescente, com a mortalidade reduzindo para 20,6% (IC95% -58,8; -6,4; p-valor 0,010) ao ano. 

A análise espacial da incidência mostrou que 37,5% dos municípios do estado apresentaram pelo menos um caso de leptospirose durante o período (Figura 2A). Destacaram-se Fortaleza (70,8%), Várzea Alegre (9,0%) e Pacoti (6,5%), que, juntos, somaram mais de 85,0% desse total. Após a suavização dos dados utilizando-se o método bayesiano empírico local (Figura 2B), observou-se uma distribuição mais homogênea da incidência dos casos estudados, de modo a serem destacados mais alguns municípios, além dos já citados: Guaramiranga, Pereiro, Ereré e Granjeiro.

A técnica Getis-Ord Gi* (Figura 2C) identificou duas regiões de áreas quentes quanto à incidência no estado. A primeira localizava-se entre a região do Maciço de Baturité e o Sertão do Cariri, contando com os seguintes municípios: Caridade, Palmácia, Guaramiranga, Baturité e Redenção. A segunda região localizava-se no Vale do Jaguaribe, com Pereiro, Iracema e Potiretama. Além disso, foi possível observar uma área quente em Granjeiro, que pertence à Região do Cariri. A varredura (Figura 2D) permitiu identificar municípios com os maiores riscos quanto à incidência de leptospirose em todo o espaço-tempo que corresponde à análise. Nesse contexto, destacaram-se: Pacoti, Ereré e Várzea Alegre. Do total, 166 municípios apresentaram RR<1.

A análise espacial mostrou que 17,5% dos municípios do estado apresentaram no mínimo um óbito quanto à leptospirose (Figura 3A), com destaque para Fortaleza (66,4%) e Caucaia (5,6%). Após suavização (Figura 3B), notou-se uma distribuição mais homogênea da mortalidade, de modo a destacar grandes áreas abrangendo Fortaleza (capital) e sua região metropolitana, além de outras, referentes à Serra da Ibiapaba (Oeste do estado) e ao Vale do Jaguaribe (Leste do estado). 

A técnica Getis-Ord Gi* (Figura 3C) identificou as principais áreas quentes quanto à mortalidade. A principal correspondeu aos municípios entre o Sertão do Canindé (Canindé, Paramoti e Caridade), o Litoral Oeste (General Sampaio, Apuiarés e Tejuçuoca) e o Maciço de Baturité (Aratuba, Capistrano e Mulungu). Outras áreas quentes isoladas foram encontradas em Pereiro, Moraújo, Pacujá, Maranguape, Palmácia e Chorozinho. A varredura (Figura 3D) para risco relativo identificou sete municípios no espaço-tempo analisado que possuíam RR>1: Fortaleza, Palmácia, Aratuba, Tururu, General Sampaio, Moraújo e Graça. Os outros 154 municípios do estado apresentaram RR<1.

**Figura 2:** Distribuição espacial anual da incidência por leptospirose: (A) análise de taxa bruta, (B) após suavização bayesiana empírica local, (C) por Getis-Ord Gi* e (D) risco relativo (RR). Ceará, 2007-2023 (n=1.319)

**Figura 3:** Distribuição espacial anual da mortalidade por leptospirose: (A) análise de taxa bruta, (B) após suavização bayesiana empírica local, (C) por Getis-Ord Gi* e (D) risco relativo (RR). Ceará, 2007-2023 (n=143)

Por fim, identificaram-se aglomerados espaço-temporais quanto à incidência e à mortalidade da doença no estado. Investigando a incidência (Figura 4A), evidenciou-se Icó como o principal aglomerado do Ceará, com RR 27,2 no período 2008-2009 (p-valor<0,001). Outros aglomerados significativos foram encontrados em: Guaramiranga, no período 2007-2014 (RR 66,2; p-valor<0,001), Fortaleza, entre 2007 e 2012 (RR 3,1; p-valor<0,001), e Várzea Alegre, no período 2016-2022 (RR 2,3). 

A análise identificou outros quatro aglomerados no período, porém não significativos: Várzea Alegre, em 2011-2011 (RR 7,8; p-valor 0,078), Farias Brito, entre 2018 e 2022 (RR 5,6; p-valor 0,340), Independência, em 2012-2012 (RR 17,8; p-valor 0,711), e Sobral, em 2022-2022 (RR 5,1; p-valor 0,790). Já quanto à mortalidade (Figura 4B), encontraram-se apenas duas observações, ambas não significativas, em Caucaia, entre 2016 e 2022 (RR 5,1; p-valor 6,704), e Paracuru, entre 2007 e 2011 (RR 2,2; p-valor 0,852). Todos os municípios descritos nos resultados podem ser consultados na Figura Suplementar 2.

## Discussão 

O perfil dos casos de leptospirose identificados no Ceará reflete um padrão já reconhecido em diferentes contextos, tanto no Brasil quanto em outros países, reforçando que certos grupos populacionais estão sistematicamente mais expostos à doença. A expressiva concentração de casos em homens adultos, com baixa escolaridade e de raça/cor da pele parda ou branca revela como os fatores sociais, econômicos e ocupacionais estão relacionados à exposição à leptospirose ^2^^,^^13^^-^^15^. 

Esses indivíduos frequentemente atuam em atividades laborais que exigem contato com ambientes insalubres ou expostos a enchentes e a áreas alagadas, como ocorre com trabalhadores agrícolas, médicos veterinários, caçadores, viajantes e militares ^16^. Assim, trata-se de uma doença marcada por desigualdades estruturais, em que o trabalho, a escolaridade e o pertencimento racial ou étnico moldam o nível de exposição e de adoecimento por leptospirose. 

O achado sobre o comportamento temporal da incidência segue um padrão semelhante ao observado em estudos realizados em Santa Catarina (região Sul) que, assim como esta pesquisa, utilizaram análise de séries temporais com dados secundários. Embora o contexto climático desse estado seja distinto em relação ao Ceará, com pluviosidade mais contínua o ano todo ^17^, esse achado reforça o papel central das chuvas na dinâmica da doença, de acordo com a literatura específica ^12^^,^^18^^,^^19^.

Há poucos estudos que investigam a distribuição espacial da incidência da doença no Ceará. Entretanto, evidencia-se que os municípios do estado que necessitam de mais atenção não são considerados grandes centros urbanos. Por exemplo, Pacoti, Pereiro e Várzea Alegre apresentaram alta incidência após suavização bayesiana e risco acima da média, o que pode estar associado a um histórico de surto da doença ocorrido há cerca de dez anos ^20^^,^^21^. 

A análise espacial da incidência com e sem suavização bayesiana evidencia diferenças relevantes na identificação dos municípios prioritários. No caso de Pacoti, Pereiro e Várzea Alegre, observa-se que a suavização não apenas manteve a alta incidência previamente identificada, como também ressaltou o risco elevado em áreas adjacentes. Tal comportamento pode estar associado ao pequeno porte populacional desses municípios, o que torna suas taxas mais suscetíveis a flutuações por número reduzido de casos. Ademais, regiões rurais também tendem a se destacar quanto à leptospirose, devido a uma maior exposição a áreas com sinais de roedores, a locais de criação de animais e de armazenamento de grãos, além da proximidade a rios e córregos ^22^.

**Figura 4:** Identificação anual de aglomerados, períodos e risco relativo (RR): (A) incidência e (B) mortalidade por leptospirose. Ceará, 2007-2023 (n=1.319 para incidência; n=143 para mortalidade)

Áreas urbanas, por sua vez, possuem outros eventos de exposição à doença, tais como: contato com fossas sépticas, proximidade a terrenos baldios, acúmulo de lixo, contato direto com roedores, fontes inadequadas de água para consumo e inundações ^14^^,^^23^. A identificação de aglomerados de incidência revelou também predominância em municípios do interior, caracterizados por populações pequenas.

No entanto, o achado que aponta Fortaleza, capital e cidade de maior população do estado, e Sobral, a quinta maior em termos populacionais, como os principais aglomerados estão em consonância com as pesquisas atuais. Essas destacam os grandes centros urbanos como os maiores contribuintes para o quantitativo geral de casos de leptospirose. Nessas áreas, as vulnerabilidades urbanas, como descarte inadequado de resíduos sólidos, falhas na coleta de lixo e contaminação de recursos hídricos desempenham um papel crucial na propagação da bactéria no ambiente. Esses fatores são intensificados durante períodos de chuvas intensas, favorecendo o aumento dos casos. Estudo realizado em Fortaleza identificou aumento expressivo na incidência de doenças relacionadas ao manejo inadequado do lixo, incluindo a leptospirose, reforçando a relevância desse contexto urbano para a transmissão da doença ^24^.

Quanto à mortalidade, sua evolução temporal é pouco debatida na literatura no Ceará. Embora pesquisas indiquem uma tendência de redução dos óbitos pela doença em âmbito nacional e internacional ^2^^,^^25^^-^^27^, observa-se neste estudo uma dinâmica mais complexa no Ceará, com períodos distintos de aumento e de diminuição da mortalidade. Além disso, os achados relativos à mortalidade reforçam padrões já observados na incidência, com maior ocorrência entre homens, pessoas com baixa escolaridade e em contextos de vulnerabilidade. Fatores ambientais, como elevada pluviosidade e ocorrência de enchentes, também se associam de forma significativa aos óbitos por leptospirose, contribuindo para a gravidade dos desfechos ^2^^,^^14^^,^^25^^-^^27^.

A análise espacial destaca Fortaleza como o município com maior risco para mortalidade pela doença no estado e Caucaia como o principal aglomerado. Salienta-se que não se identificaram outros estudos na literatura que avaliaram a distribuição espacial da doença no Ceará, portanto comparações não são possíveis. Entretanto, estudos nacionais conduzidos em outros estados, como no Rio Grande do Sul, entre 2007 e 2019, também identificaram a concentração de óbitos e o maior risco de contágio em capitais e regiões metropolitanas. Foram evidenciados aglomerados de alta incidência e risco em áreas urbanas mais densamente povoadas, corroborando os achados desta pesquisa, o que evidencia a associação entre vulnerabilidades urbanas e a maior mortalidade por leptospirose ^28^.

Além da pluviosidade, outros estudos, que avaliam a dinâmica espacial da mortalidade por leptospirose, reforçam a influência da vulnerabilidade socioeconômica ^29^, embora salientem ^5^ que determinantes causais são mais bem visualizados na incidência e pouco o são na mortalidade. Assim, pesquisas sobre leptospirose devem considerar as desigualdades sociais, ambientais e de acesso à saúde como fatores contribuintes ao adoecimento ^30^, pois são deficiências que prejudicam o enfrentamento da doença e favorecem a negligência diante do seu manejo.

O estudo revelou variações na incidência e na mortalidade por leptospirose ao longo do período analisado, com fases alternadas de aumento e de redução, sem evidência de tendência geral de queda sustentada. De um lado, a concentração de casos em municípios do interior do estado, como Pacoti, Pereiro e Várzea Alegre, reflete a influência de condições ambientais e socioeconômicas em municípios que não são considerados centros urbanos. Por outro lado, as maiores taxas de mortalidade registradas na capital, Fortaleza, e em Caucaia, município da região metropolitana, ressaltam as fragilidades de áreas densamente povoadas e agravadas por episódios de intensas chuvas. 

Sendo assim, a dinâmica temporal indica influência de fatores ambientais e sazonais, enquanto a distribuição espacial revela que o risco não se limita apenas aos centros urbanos, alcançando municípios interioranos também marcados por vulnerabilidades persistentes. Nesse contexto, destacam-se algumas limitações deste estudo, sobretudo a utilização de bases de dados secundários, suscetíveis à incompletude ou à não aderência por profissionais de saúde ao preenchimento da ficha de notificação da doença. Portanto, tais achados são sensíveis a uma falácia ecológica. Além disso, os dados populacionais podem não corresponder, com fidelidade, ao real. Desse modo, os autores destacam que tais achados são apenas estimativas. Ressalta-se também que a utilização de taxas bayesianas pode causar dependência espacial nos dados, pois as características do método dependem da máxima verossimilhança. Logo, devem ser interpretados com cautela.

Assim sendo, conclui-se que a leptospirose no Ceará apresenta um padrão espaço-temporal heterogêneo, condicionado por fatores sociais, ambientais e estruturais. Esses achados reforçam a necessidade de medidas integradas de prevenção e de controle, como ampliação da infraestrutura sanitária, monitoramento das áreas de maior risco, controle de roedores e ações de educação em saúde. O fortalecimento da vigilância epidemiológica e a priorização de políticas públicas nas regiões mais críticas são fundamentais para reduzir a carga da leptospirose e proteger as populações mais vulneráveis

## Data Availability

Os dados utilizados na pesquisa foram organizados na plataforma Open Science Framework, acessível por meio do link: https://osf.io/h7s8z/?view_only=89a03da727e84108a642b5a3e64c54ef.

## Material Suplementar

Figura Suplementar
